# Combination of ruthenium(II)-arene complex [Ru(η^6^-*p*-cymene)Cl_2_(pta)] (RAPTA-C) and the epidermal growth factor receptor inhibitor erlotinib results in efficient angiostatic and antitumor activity

**DOI:** 10.1038/srep43005

**Published:** 2017-02-22

**Authors:** Robert H. Berndsen, Andrea Weiss, U. Kulsoom Abdul, Tse J. Wong, Patrick Meraldi, Arjan W. Griffioen, Paul J. Dyson, Patrycja Nowak-Sliwinska

**Affiliations:** 1 Angiogenesis Laboratory, Department of Medical Oncology, VU University Medical Center, Amsterdam, The Netherlands; 2 Institute of Chemical Sciences and Engineering, Swiss Federal Institute of Technology (EPFL), Lausanne, Switzerland; 3 Department of Cell Physiology and Metabolism, University of Geneva Medical School, University of Geneva (UNIGE), Geneva, Switzerland; 4 School of Pharmaceutical Sciences, University of Geneva (UNIGE), Geneva, Switzerland

## Abstract

Ruthenium-based compounds show strong potential as anti-cancer drugs and are being investigated as alternatives to other well-established metal-based chemotherapeutics. The organometallic compound [Ru(η^6^-*p*-cymene)Cl_2_(pta)], where pta = 1,3,5-triaza-7-phosphaadamantane (RAPTA-C) exhibits broad acting anti-tumor efficacy with intrinsic angiostatic activity. In the search for an optimal anti-angiogenesis drug combination, we identified synergistic potential between RAPTA-C and the epidermal growth factor receptor (EGFR) inhibitor, erlotinib. This drug combination results in strong synergistic inhibition of cell viability in human endothelial (ECRF24 and HUVEC) and human ovarian carcinoma (A2780 and A2780cisR) cells. Additionally, erlotinib significantly enhances the cellular uptake of RAPTA-C relative to treatment with RAPTA-C alone in human ovarian carcinoma cells, but not endothelial cells. Drug combinations induce the formation of chromosome bridges that persist after mitotic exit and delay abscission in A2780 and A2780cisR, therefore suggesting initiation of cellular senescence. The therapeutic potential of these compounds and their combination is further validated *in vivo* on A2780 tumors grown on the chicken chorioallantoic membrane (CAM) model, and in a preclinical model in nude mice. Immunohistochemical analysis confirms effective anti-angiogenic and anti-proliferative activity *in vivo,* based on a significant reduction of microvascular density and a decrease in proliferating cells.

Metal-based chemotherapeutics including cisplatin, carboplatin and oxaliplatin have been used routinely in the clinic for decades and continue to represent the mainstay of treatment for many cancer types^1^. Despite the clinical success of platinum-based chemotherapeutics, their ability to prolong overall patient survival is often restricted by dose-limiting side effects, as well as intrinsic and acquired resistance^2^. Consequently, significant research efforts have focused on developing compounds based on other metals^3^.

In recent years, there has been particular interest in the development of ruthenium-based drugs and several have been shown to exhibit clear anti-cancer activity *in vitro* and anti-tumor activity in various animal models^4^,^5^,^6^. Moreover, Ru-based compounds generally exhibit superior toxicity profiles compared to platinum-based compounds^2^,^6^. The Ru(III) compounds, KP1019 [indazolium trans-[tetrachlorobis(1H-indazole)ruthenate(III)]]^7^,^8^,^9^, KP1339 (the water soluble sodium salt of KP1019)^10^,^11^ and NAMI-A [imidazolium trans-[tetrachloro(dimethylsulfoxide)(1H-imidazole)ruthenate(III)]]^12^ have completed phase I and phase I/II trials, for NAMI-A in combination with gemcitabine^13^, and are expected to undergo further clinical evaluation. In addition to Ru(III) compounds, a number of promising Ru(II)-based compounds have been evaluated various *in vivo* models^14^. For example, [Ru(η^6^-*p*-arene)Cl(en)]^+^(arene = *p*-cymene or biphenyl, en = ethylenediamine) was shown to reduce the growth of primary tumors^4^,^15^.

The compound [Ru(η^6^-*p*-cymene)Cl_2_(pta)], where pta = 1,3,5-triaza-7-phosphaadamantane (RAPTA-C) was originally only shown to have anti-metastatic properties^16^ and, subsequently, was shown to exhibit primary anti-cancer effects by inducing apoptosis in Ehrlich ascites carcinoma cells^17^. While the mechanism of action of RAPTA-C still remains to be fully elucidated, it has been suggested that the formation of adducts in chromatin histone proteins are involved^18^. This mechanism is different to that observed for the ruthenium(III) compounds mentioned above^19^,^20^,^21^. We have also demonstrated the effect of RAPTA-C on the suppression of primary tumor growth in preclinical studies^22^. In addition to targeting cancer cells directly, strong anti-angiogenic activity has been demonstrated both *in vitro* and *in vivo*^23^.

Extensive studies have been undertaken to modify the structure of Ru(II) compounds to improve their drug efficacy^24^,^25^, while much less effort has been directed towards elucidating drug combinations^13^,^26^, despite the latter approach being well-established in enhancing therapeutic efficacy^27^,^28^. For instance, targeted therapies have been shown to enhance the effect of classical chemotherapy by reducing the likelihood of acquired drug resistance resulting in prolonged patient survival^29^,^30^. However, only few drug combination studies investigating metal-based drugs that do not contain platinum have been performed. For instance, combinations of NAMI-A and 5-fluorouracil, cisplatin or doxorubicin showed superior activity compared to either chemotherapeutic alone in various pre-clinical cancer models^31^,^32^,^33^. However, in a recent phase I/II trial in NSCLC patients, NAMI-A and gemcitabine did not show enhanced activity^13^. Furthermore, another Ru-based compound, KP1339, was recently found to synergistically improve the effect of the multi-kinase inhibitor sorafenib in hepatoma cancer *in vitro* and *in vivo*^26^.

In a recent study employing an algorithm-based screening method incorporating a broad selection of targeted drugs, we optimized an angiostatic drug combination for the treatment of cancer^34^. This study hinted towards dose-dependent synergistic interactions between RAPTA-C and the epidermal growth factor receptor (EGFR) inhibitor, erlotinib/Tarceva^®^ in various *in vitro* and *in vivo* models^34^. Erlotinib is a small molecule tyrosine kinase inhibitor (TKI) targeting EGFR and with lower affinity also targeting serine/threonine kinases (i.e. cyclin G-associated kinase, serine/threonine-protein kinase 10 and STE20-like serine/threonine-protein kinase)^35^. It is currently approved for the treatment of non-small cell lung cancer and for the treatment of pancreatic cancer in combination with gemcitabine^36^. Erlotinib competes with ATP binding to the tyrosine kinase domain of EGFR and has been shown to act through the inhibition of cell proliferation and the induction of cell cycle arrest in cancer cells^37^,^38^. Importantly, through the blockage of EGFR and its downstream ras/raf/MEK/MAPK signalling pathway, erlotinib also inhibits the release of pro-angiogenic factors, including vascular endothelial growth factor (VEGF), interleukin 8 (IL8) and fibroblast growth factor (FGF)^39^,^40^. As erlotinib and RAPTA-C both act through anti-cancer and anti-angiogenic mechanisms, their combination might be beneficial in the treatment of aggressive tumor types.

For the current study, we undertook a detailed evaluation of the therapeutic potential of the erlotinib/RAPTA-C combination by identifying effective drug dose ratios and studying the mechanism of action of this drug combination. Studies were performed *in vitro* using endothelial and human A2780 ovarian carcinoma cells, as well as in A2780 cells with acquired resistance to cisplatin (A2780cisR). The *in vitro* experiments were subsequently validated *in vivo* using the chicken chorioallantoic membrane (CAM) model grafted with A2780 or A2780cisR tumors, and in nude mice bearing A2780 tumors. The results presented here show the effective activity of these two compounds when administered simultaneously, leading to effective tumor growth inhibition.

## Results

### Cell viability and migration assays

**Figure 1:**
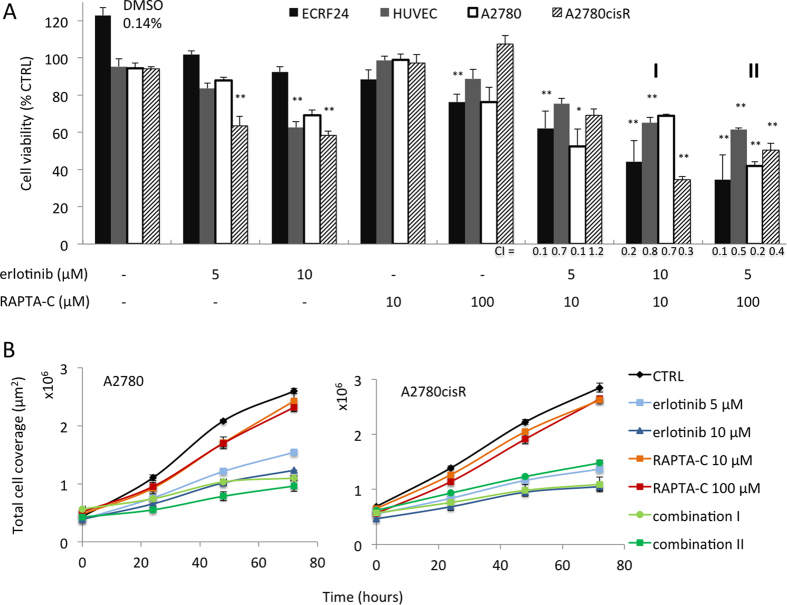
Activity of erlotinib and RAPTA-C on endothelial and ovarian cancer cell viability (**A**) and on total cell coverage per μm^2^ surface (**B**). (**A**) Inhibition of cell viability by erlotinib, RAPTA-C and their combinations in endothelial cells (immortalized ECRF24 and primary human umbilical vein endothelial cells; HUVEC) and ovarian cancer cells (A2780 and A2780cisR). The combination of erlotinib 5 μM/RAPTA-C 10 μM, erlotinib 10 μM/RAPTA-C 10 μM (marked as **I)** and erlotinib 5 μM/RAPTA-C 100 μM (marked as **II**) are shown. Cell viability was assessed after 72 h of drug treatment by the CellTiter-Glo luminescence assay and represented as a percentage of the control. Combination index (CI) values per cell line for erlotinib/RAPTA-C combinations are shown. Significance is indicated vs. CTRL (0.14% DMSO-treated cells), with *P < 0.05 and **P < 0.01, based on a one-way ANOVA with post-hoc Tukey’s test (ECRF24: F(19, 219) = 13.69, P < 0.0001; HUVEC: F(19, 175) = 16.83, P < 0.0001; A2780: F(19, 85) = 10.86, P < 0.0001; A2780cisR: F(19, 205) = 58.2, P < 0.0001). (**B**) Total cell coverage of A2780 and A2780cisR cells attached to the bottom of a 96-well plate. Total cell coverage was assessed at 0, 24, 48 and 72 hours of drug treatment and automatically measured and quantified. Values shown represent absolute values of the total surface in μm^2^ occupied by cells. Values represent the mean of at least one experiment performed in triplicate and error bars represent the SEM.

The effect of erlotinib and RAPTA-C on cell viability was investigated in immortalized (ECRF24) and primary (HUVEC) human endothelial cells (ECs), as well as in human A2780 ovarian carcinoma cells and a cisplatin-resistant variant of this cell line, A2780cisR (Fig. 1A). Dose response curves for both compounds applied as mono-therapies were previously reported for the ECRF24 cell line^34^ and were prepared for the other cell lines (data not shown). We selected a dose range that inhibits cell viability by ca. <40% based on these curves (for erlotinib <15 μM and for RAPTA-C <200 μM). Notably, combinations of erlotinib/RAPTA-C significantly inhibited cell viability (erlotinib 10 μM/RAPTA-C 10 μM, marked as combination **I**, and erlotinib 5 μM/RAPTA-C 100 μM, marked as combination **II**; Fig. 1A and Supplementary Figure 1 for other investigated dose ratios). Measurement of absolute cell numbers in A2780 and A2780cisR cells showed that the cell count for erlotinib/RAPTA-C treated cells did not increase much (indicative of halted cell proliferation) whereas the cell count of non-treated cells tripled after 72 hours (Fig. 1B). This difference suggests that erlotinib/RAPTA-C combinations induce a state of cellular senescence, as the cell number also does not decrease (which would be suggestive of cell death). Interestingly, assessment of A2780 and A2780cisR cell counts closely resembled the activity on cell viability at 24, 48 and 72 hours of treatment (Supplementary Figure 2). To assess specificity and potential toxicities, the activity of erlotinib and RAPTA-C was tested in the non-malignant cells types human embryonic kidney cells and peripheral blood mononuclear cells (HEK-293 and PBMCs respectively; Supplementary Figure 3). PBMC and HEK cell viability was only minimally affected by either erlotinib, RAPTA-C or their combinations, suggesting increased specificity towards activated endothelial cells and cancer cell types, and a favourable toxicity profile of the drug combination.

To investigate possible synergies between erlotinib and RAPTA-C, the combination index (CI) was determined for each drug combination (Fig. 1A and Supplementary Table 1). A CI < 1 indicates a synergistic effect, when CI = 1 the effect is additive and, a CI > 1 corresponds to antagonism. Synergistic efficacy (CI < 1) was observed for nearly all of the combinations tested in all cell types.

The effect of varying drug administration schedules on the overall efficacy of drug combinations in the ECRF24 and A2780 cell lines *in vitro* was additionally investigated. Thus, the efficacy of each combination was examined when adding one drug 2 h prior to the addition of the other drug, and *vice versa*. A comparison of both dosing schedules on cell viability inhibition revealed no significant differences that could be attributed to the administration schedule (data not shown).

We further evaluated the activity of erlotinib/RAPTA-C combinations on their ability to inhibit the mobility of ECRF24 (endothelial-), A2780 and A2780cisR (cancer-) cells using the scratch wound assay. Scratches were made in a confluent cell layer and relative wound closure was determined. Neither single drug nor any of the evaluated drug combination inhibited the mobility of the ECRF24 cells significantly, which is in concordance with results from our previous study^34^. It should be noted that in the scratch wound assay, cells are only treated for a period of 6 h, whereas cell viability is assessed after a 72 h period of exposure to the compounds, which may partially account for the limited activity observed in the scratch wound assay (Supplementary Figure 1).

**Figure 2:**
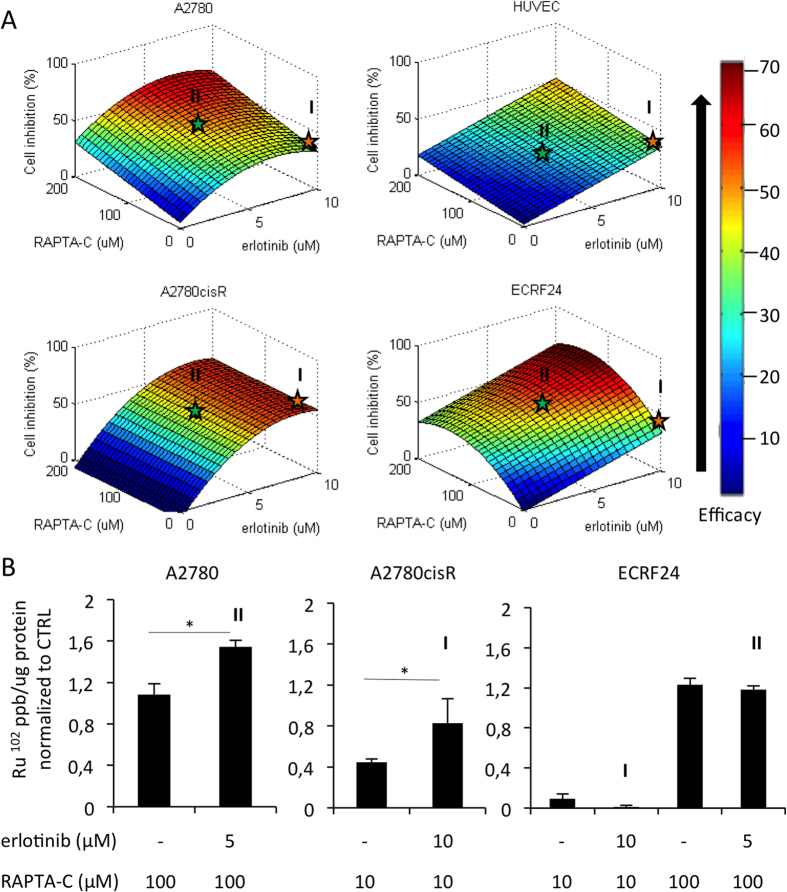
Response surfaces of ovarian cancer- and endothelial cell viability inhibition with respect to varying doses of erlotinib/RAPTA-C in combination treatments and cellular uptake of Ru in ovarian cancer cells. (**A**) Images show a visual representation of the effect, in terms of cell viability inhibition, of systematically varying the drug concentrations comprising erlotinib and RAPTA-C combinations. A range is shown from low efficacy (blue) to high efficacy (red). The orange star represents combination **I**; erlotinib/RAPTA-C (10/10 μM) and the green star represents combination **II**; erlotinib/RAPTA-C (5/100 μM). These dose-ratios were selected for translation to *in vivo* experiments. The input data used to generate the response surfaces are shown in Fig. 1A and Supplementary Figure 1. (**B**) Cellular uptake of Ru in each cell line for the optimal combination identified in cell proliferation assays. Values represent the mean Ru^102^ signal parts per billion (ppb) normalized to the protein content (per μg of protein) of each sample from one experiment performed in triplicate with error bars representing the SEM. Significance is indicated for combination treatment vs. the RAPTA-C single drug treatment group (*P = 0.028 and 0.045 in A2780 and A2780cisR cells, respectively; *P < 0.05 and **P < 0.01).

To visualize the relationship between varying concentrations of erlotinib/RAPTA-C and the drug combination efficacy (in terms of cell viability inhibition), response surfaces were constructed for each cell type. The shape of the response surface differs between cell types (Fig. 2A). Response surfaces were used to identify the combinations to be used in the *in vivo* models. The starred dose ratio combinations (erlotinib 10 μM/RAPTA-C 10 μM, marked as **I**, and erlotinib 5 μM/RAPTA-C 100 μM, marked as **II** in Fig. 2A), were selected for translation to *in vivo* testing as they represent the minimal drug dosages within the area of the response surface showing maximal efficacy (red/orange region of the surface), for A2780cisR (combination **I**) and A2780 (combination **II**). Moreover, both combinations result in similar inhibition of the endothelial cell types HUVEC and ECRF24, thus supporting an anti-proliferative effect of both combinations in endothelial cell types.

### Cellular ruthenium uptake

It was previously reported that intracellular accumulation of Ru may account for synergistic interactions between KP1339 and sorafenib^26^. To investigate a possible similar mechanism underlying the observed synergies, the cellular uptake of ruthenium was analysed using inductively coupled plasma mass spectrometry (ICP-MS) in each cell line following a 5 h exposure period to the selected, optimal drug combination or the corresponding RAPTA-C dose alone (Fig. 2B). Ru accumulates in the cancer cells at both 10 and 100 μM concentrations of RAPTA-C (1.08 mean Ru^102^ signal (ppb/μg of protein) at 100 μM in A2780 and 0.45 (ppb/μg of protein) at 10 μM in A2780cisR). Furthermore, erlotinib enhances the uptake of Ru in both cancer cell lines significantly as compared to RAPTA-C administered alone (i.e. erlotinib induced increase in mean Ru^102^ signal of 0.46 (ppb/μg of protein) in A2780 and 0.38 (ppb/μg of protein) in A2780cisR; Fig. 2B).

RAPTA-C at a 10 μM dose is not observed to accumulate in ECRF24 cells, whereas exposure to 100 μM of RAPTA-C results in significant Ru uptake (mean Ru^102^ signal 0.09 (ppb/μg of protein) and 1.23 (ppb/μg of protein) for RAPTA-C 10 μM and 100 μM, respectively). The addition of erlotinib to these RAPTA-C concentrations did not change the Ru uptake in ECRF24 cells for either drug combination (mean Ru^102^ signal 0.01 (ppb/μg of protein) and 1.18 (ppb/μg of protein) for combination **I** and **II**, respectively).

### Mechanism of cell death induced by erlotinib/RAPTA-C combinations

**Figure 3:**
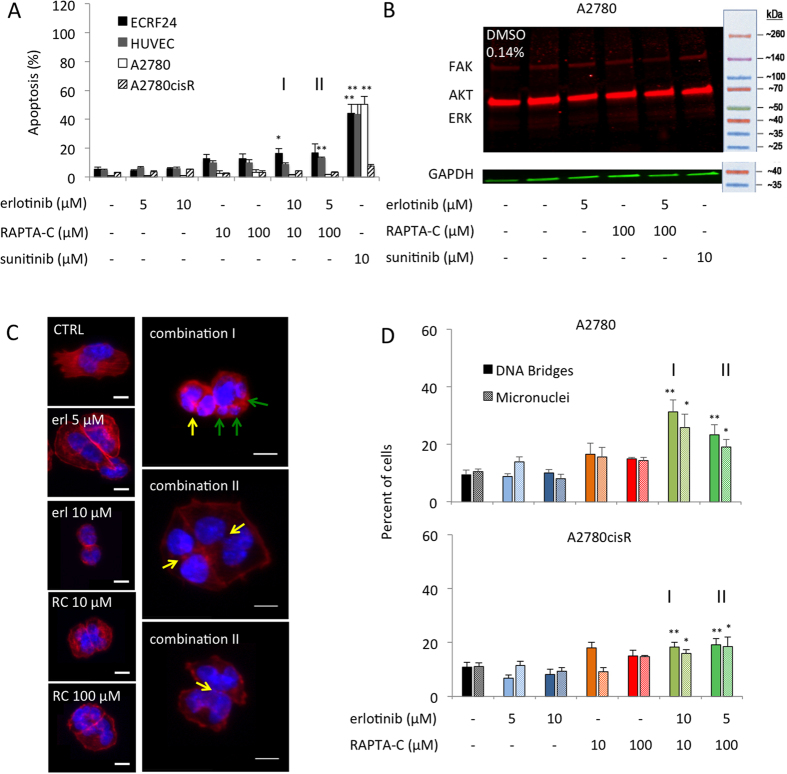
Apoptosis induction in human endothelial- and ovarian carcinoma cells, and the formation of DNA bridges and micronuclei in ovarian carcinoma cells. Apoptosis and cell cycle distributions were assessed using flow cytometry analysis based on propidium iodide (PI) staining of DNA. Sunitinib (10 μM) was used as a positive control. The combination consisting of erlotinib 10 μM/RAPTA-C 10 μM is marked as **I** and erlotinib 5 μM/RAPTA-C 100 μM is marked as **II**. (**A**) Apoptosis induction in ECRF24, HUVEC, A2780 and A2780cisR cells. (**B**) Western Blot analysis of erlotinib/RAPTA-C treatment in A2780 cells. Cells were treated for 2 h after which cell lysates were prepared. Immunoblotting was performed using FAK, AKT and ERK antibodies. GAPDH was included as a loading control. *P < 0.05, **P < 0.01 indicate significance vs. the control. (**C**) Representative images of staining for DAPI (blue) and phalloidin (red) in A2780 cells. The yellow arrows indicate DNA bridges and the green arrows indicate micronuclei. (**D**) Quantification of DNA Bridges between nuclei and quantification of micronuclei in DAPI stained A2780 cells. Statistical analysis was performed with a one-way ANOVA (A2780 bridges: F (6, 29) = 4.739, P = 0.0018; A2780 micronuceli: F (6, 29) = 3.293, P = 0.0135; A2780cisR bridges: F (6, 28) = 8.650, P > 0.0001; A2780cisR micronuceli: F (6, 28) = 2.469, P = 0.0482). Values represent the mean of at least one experiment performed in triplicate and error bars represent the SEM.

To investigate the mechanism of cell death of erlotinib/RAPTA-C combinations in ECs and ovarian carcinoma cells, DNA profiles were assessed by flow cytometry analysis after staining with propidium iodide (PI). Sunitinib (10 μM) was included in the assays as a positive control for apoptosis^41^. While the single drug treatments did not induce significant apoptosis, combination **I** and **II** lead to the induction of apoptosis (e.g. 16.3% and 16.7% apoptosis induction in ECRF24 cells for combination **I** and **II**, respectively, Fig. 3A). Interestingly, in both A2780 and cisplatin-resistant A2780 (A2780cisR) cells, only a minor induction of apoptosis was observed (Fig. 3A and Supplementary Figure 4A for other investigated dose ratios).

To further explore the mechanism of the synergistic anti-proliferative activity of erlotinib/RAPTA-C combinations the cell cycle distribution was analysed. In all the cell types tested, no clear G2/M- or S-phase arrest was observed for erlotinib, RAPTA-C or their combinations compared to controls (shown for ECRF24 cells in Supplementary Figure 4B). Furthermore, the differences in S-phase, G0/G1 and G2/M phases for all the conditions tested were only marginally (not significantly) distinct compared to the controls. These data indicate that apoptosis induction contributes to the mechanism by which the erlotinib/RAPTA-C combination induces cell death in ECRF24 and HUVEC cells, but not in A2780 and A2780cisR cells. In addition, in both A2780 and A2780cisR cells, the induction of autophagy was studied since the erlotinib/RAPTA-C treatment did not result in clear apoptosis induction and there is evidence suggesting that the induction of autophagy is an alternative route to cell death^42^. However, erlotinib/RAPTA-C treatment did not result in an increase in the formation of autophagic vacuoles in either cell lines (Supplementary Figure 5).

In the next step, we controlled whether treatment with combination II affected the expression of FAK (Fig. 3B), known to be enhanced in human ovarian cancer and linked with ovarian carcinoma dissemination^43^. The immunoblotting results revealed attenuated FAK expression by combination **II** in A2780 cells, which can be seen by the decreased intensity and thickness of corresponding western blot bar (Fig. 3B). However, neither phosphorylated FAK, AKT or ERK was affected by the combination treatment in A2780 cells (Supplementary Figure 6). Moreover, Rhodamine-Phalloidin staining against actin (Fig. 3C in red) revealed that treated cells appeared more rounded and showed a tendency to aggregate. Additionally, the polymerization of actin was clearly disrupted and stress fibers were decreased. The stained actin fibers in the treated cells were condensed in the cell periphery as compared to the central part of the cell in the CTRL group. Moreover, pertusions visible in CTRL cells were attenuated in combinations and monotherapy-treated cells. These phenomena were previously reported in FAK-deficient cells^44^. Levels of phosphorylated proteins were not altered (Supplementary Figure 7).

To detect potential defects in actin organization and cellular morphology, we next stained cells after 24 hours of treatment with fluorescent phalloidin (actin marker) and DAPI (DNA marker; Fig. 3C,D, blue). After treatment with combination **I** (**P < 0.01 vs. CTRL, one-way ANOVA), and to lesser extend combination **II** (**P < 0.01 vs. CTRL, one-way ANOVA), we observed in A2780 and A2780cisR cells, but not in ECRF24 cells (Supplementary Figure 6), a significant increase in the number of DNA bridges (Fig. 3C, right column, top image, yellow arrows). Such DNA-bridges arise as a consequence of entering mitosis with partially unreplicated DNA, chromosome fusions or as a result of severe chromosome segregation errors^45^. These cells also frequently displayed micronuclei (Fig. 3C, right column, middle image), another marker for chromosome segregation errors or chromosome breakages, which is linked to genomic instability and chromosomal damage^46^. DNA bridges persist after mitotic exit, delay abscission and can lead to a cytokinesis failure and tetraploidization^47^. Consistent with such a hypothesis, cells with DNA bridges often contained remnants of the cytokinetic rings, implying cytokinesis failure (Fig. 3C, right column, bottom image). Thus, combinations **I** and **II** did not alter cell cycle progression, but the outcome of cell division in A2780 and A2780cisR cells.

### Evaluation of the *in vivo* anti-vascular effects of erlotinib/RAPTA-C combinations

**Figure 4:**
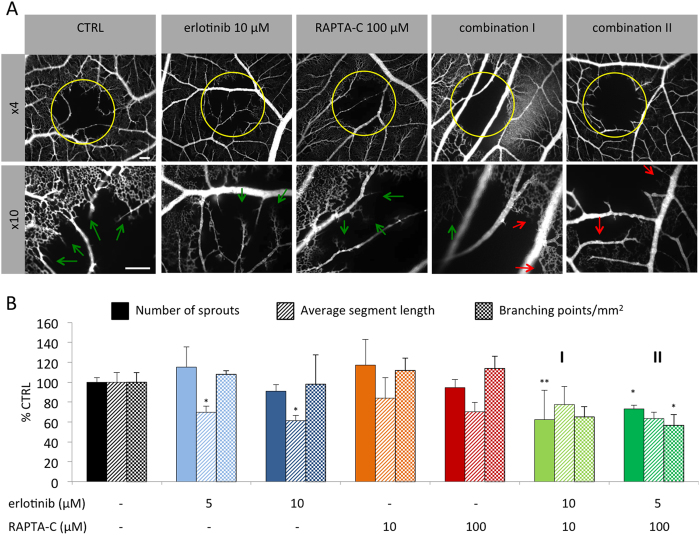
Activity of erlotinib/RAPTA-C combinations on the CAM vasculature. (**A**) Fluorescence angiogram of PDT induced neo-vascularization on the CAM after treatment with erlotinib, RAPTA-C and erlotinib/RAPTA-C combinations. The yellow circle shows the PDT-treated area with ongoing revascularization. Green arrows show vascular sprout formation as result of PDT treatment. Red arrows indicate occluded blood vessels. (**B**) Quantification of the number of sprouts, the average length of vascular sprouts and the number of branching points per mm^2^ was performed using ImageJ. Drug administration was performed directly after PDT and fluorescence angiograms were recorded 24 h after treatment. Erlotinib/RAPTA-C (10/10 μM) and erlotinib/RAPTA-C (5/100 μM) are referred to as combination **I** and **II**, respectively. The white bars represent 200 μm and are valid for all images with the corresponding magnification. N = 5–6 eggs. Values represent the mean and error bars represent SEM. Statistical analysis was achieved using a one-way ANOVA test with post-hoc Dunnett’s multiple comparison between the combination and control groups only (number of sprouts: F(2,31) = 3.51, P = 0.0415; average segment length: F(2,14) = 7.607, P = 0.0058; branching points per mm^2^: F(3,9) = 5.63, P = 0.0188). *P < 0.05 and **P < 0.01 indicate significance vs. the CTRL (0.14% DMSO).

The inhibition of new vascular sprout formation was evaluated in the chicken chorioallantoic membrane (CAM) model^48^ following vaso-occlusive Visudyne^®^-photodynamic therapy (PDT)^49^. Application of PDT to the CAM vasculature leads to occlusion of blood vessels, which results in the induction of angiogenesis and revascularization of the treated tissue^49^. This model of induced angiogenesis is used between embryo development day (EDD) 11 and 12, when neo-vascularization is complete. The activity of erlotinib/RAPTA-C combinations was investigated in this model based on the intravenous (i.v.) administration of the drugs applied directly after PDT treatment (Fig. 4A). In the control group, 24 h post-PDT, vascular sprouts (indicated by green arrows) were observed growing from the rim of the PDT-treated area towards the center of the treatment zone (Fig. 4A). In the group treated with erlotinib/RAPTA-C, a reduced number of sprouts, in addition to occluded blood vessels (indicated by red arrows) were observed. Quantification of vascular sprouts showed a significant decrease in the number of sprouts for both combinations tested (Fig. 4B; **P < 0.01 vs. CTRL for combination **I** and **P < 0.05 vs. CTRL for combination **II**). In addition, combination **II,** but not combination **I,** significantly decreased the number of branching points (i.e. the number of branches from which a sprout develops), while neither of the single drug treatments affected the number of branching points (Fig. 4B; *P < 0.05 vs. CTRL). Taken together, these results are indicative of an anti-angiogenic effect of the drug combinations.

### Inhibition of tumor growth in the CAM model by erlotinib/RAPTA-C combinations

**Figure 5:**
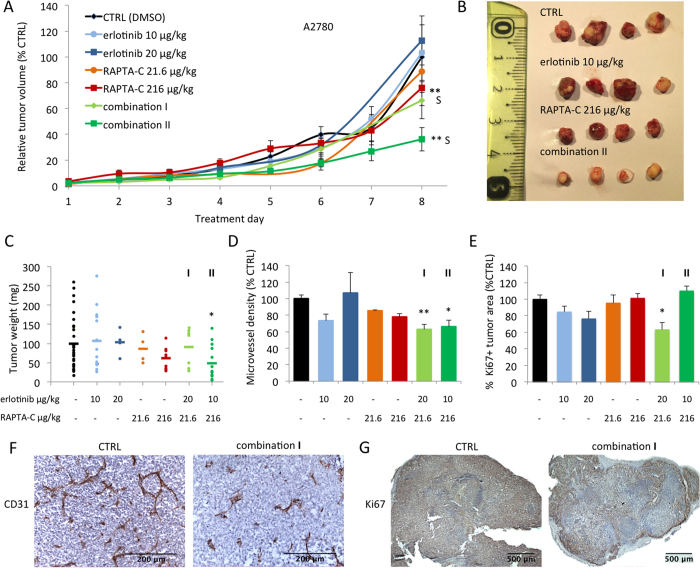
Inhibition of A2780 tumor growth in the CAM model by erlotinib, RAPTA-C and combination treatments. (**A**) Tumor growth curves of A2780 tumors grafted on the CAM showing tumor volume with respect to treatment day represented as the percentage of the final control tumor volume. S indicates synergy (CI < 1). N = 30 in the control group and N = 4–14 in the treatment groups (two-way ANOVA: F(6,555) = 8.994, P < 0.0001). (**B**) Representative images of resected tumors in each treatment group. Control tumors were treated with 0.14% DMSO in NaCl. (**C**) Tumor weight (mg) after resection on treatment day 8 (when the experiment was terminated (one-way ANOVA: F(2, 48) = 3.35, P = 0.04)). (**D**) Microvessel density (MVD) analysis measured as the number of vessels per mm^2^ of vascularized tumor area and represented as percentage of control (one-way ANOVA: F(2, 25) = 15.23, P < 0.0001). (**E**) Quantification of the percentage of whole tumor sections that are positive for the proliferation marker Ki67 (one-way ANOVA: F(2, 27) = 4.25, P = 0.025) (**F**) Representative images of immunohistochemical (IHC) staining for the endothelial cell marker CD31 (brown) counter-stained with haematoxylin (purple/blue). (**G**) Representative images of IHC staining for the proliferation marker Ki67 (brown/orange) counter-stained with haematoxylin (purple/blue). **I** indicates the combination erlotinib 20 μg/kg/day + RAPTA-C 21.6 μg/kg/day and **II** indicates the combination erlotinib 10 μg/kg/day + RAPTA-C 216 μg/kg/day in all graphs. Error bars represent SEM and *P < 0.05, **P < 0.01 indicate significance versus CTRL. Statistical analysis was performed using a two-way ANOVA with post-hoc Tukey’s multiple comparison test (**A**) or one-way ANOVA with post-hoc Dunnett’s multiple comparison test between the combination and control (**C**–**E**).

The effect of selected erlotinib/RAPTA-C combinations was investigated on the growth of both human A2780 tumors and their cisplatin resistant counterparts (A2780cisR) in the CAM model. In this model, fertilized chicken eggs were incubated in a hatching incubator and on embryo development day (EDD) 8, tumor cells were implanted onto the chorioallantoic membrane after which vascularized three-dimensional tumors appeared by EDD11. In the translation of drug doses from *in vitro* to *in vivo,* the drug dose-ratios were maintained while taking into account the suboptimal single drug efficacies previously tested (erlotinib 5 μM and 10 μM *in vitro* correspond to 10 μg/kg/day and 20 μg/kg/day *in vivo*; RAPTA-C 10 and 100 μM *in vitro* correspond to 21.6 and 216 μg/kg/day *in vivo*)^34^. Tumors were treated by 20 μl i.v. injections on treatment days 1 and 2 (EDD 11/12) and drug doses were adjusted based on the average chicken embryo weight on EDD 11/12. It was found that treatment of A2780 tumors with single drugs alone inhibited tumor growth by <20%, whereas the evaluated drug combinations inhibited tumor growth by 34% (combination **I**; vs. CTRL; not significant; CI = 0.73) and 64% (combination **II**; vs. CTRL; **P < 0.0001; CI = 0.34) on the last day of the experiment (Fig. 5A). These data were confirmed by measurement of tumor weight following resection (vs. CTRL; *P < 0.05; Fig. 5C). Macroscopic images of tumors resected on treatment day 8 show a clear reduction in tumor size, as well as differences in tumor morphology. Control and single drug treated tumors appear heterogeneous in size and colour while the combination treated tumors are generally less vascularized (indicated by the pale colour) and appear to be more homogeneous (Fig. 5B). The microvessel density (MVD) in treated A2780 tumors was quantified by staining for the endothelial cell marker CD31 in tumor sections. Interestingly, treatment with erlotinib (10 μg/kg/day) or RAPTA-C (216 μg/kg/day) alone resulted in a decrease in MVD (by 27% and 22%, respectively, Fig. 5D). Treatment with combinations of both compounds decreased MVD by 37% (combination **I**; **P < 0.01; Fig. 5) and by 34% (combination **II**; *P < 0.05; Fig. 5D).

The synergistic inhibition of A2780 tumor growth by erlotinib/RAPTA-C combinations can, at least in part, be attributed to the inhibition of angiogenesis based on the reduction in MVD seen in treated tumors. It is noteworthy that while combination **I** only slightly inhibited overall tumor growth as compared to single drug treatments (not significant vs. CTRL), it did result in significant inhibition of MVD (37%, **P < 0.01). This result is supported by the response surfaces provided in Fig. 2 where both combinations show a similar range of efficacy in the endothelial cell lines. However, a clear difference in activity of each combination is observed in the A2780 cell line since combination **II** has a much stronger inhibitory effect on tumor cell proliferation (58% cell viability inhibition) compared to combination **I** (31% cell viability inhibition).

**Figure 6:**
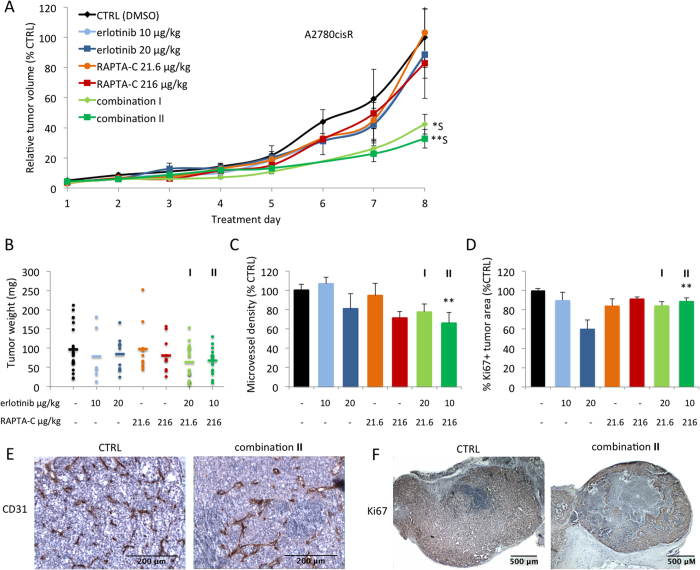
Inhibition of A2780cisR tumor growth in the CAM model by erlotinib, RAPTA-C and their combination treatment. (**A**) Tumor growth curves of A2780cisR tumors grafted on the CAM showing tumor volume with respect to treatment day, represented as the percentage of the final control tumor volume. S indicates synergy. N = 20 in the control group and N = 7–15 in the treatment groups. Control tumors were treated with 0.14% DMSO in NaCl (two-way ANOVA: F(6,550) = 4.692, P = 0.0001). (**B**) Tumor weight (mg) after resection at treatment day 8 (when the experiment was terminated; not significant, one-way ANOVA: F(2,54) = 3.05, P = 0.055). (**C**) Microvessel density (MVD) analysis measured as the number of vessels per mm^2^ of vascularized tumor area and represented as percentage of control (one-way ANOVA: F(2,31) = 5.641, P = 0.0081). (**D**) Quantification of the percentage of whole tumor surfaces that are positive for the proliferation marker Ki67 (one-way ANOVA: F(2,34) = 4.67, P = 0.016). (**E**) Representative images of IHC staining for the endothelial cell marker CD31 (brown) counter-stained with haematoxylin (purple/blue). (**F**) Representative images of IHC staining for the proliferation marker Ki67 (brown/orange) counter-stained with haematoxylin (purple/blue). **I** indicates the combination erlotinib 20 μg/kg/day + RAPTA-C 21.6 μg/kg/day and **II** indicates the combination 10 μg/kg/day + RAPTA-C 216 μg/kg/day in all graphs. Error bars represent SEM and *P < 0.05, **P < 0.01 indicate significance versus CTRL. Statistical analysis was performed using a two-way ANOVA with post-hoc Tukey’s multiple comparison test (**A**), or one-way ANOVA with post-hoc Dunnett’s multiple comparison test between the combination and control (**B**–**D**).

To assess the effect of drug treatment on the amount of proliferating cells, tumor tissues were stained for the proliferation marker Ki67. Quantification of Ki67^+^ staining in whole tumor surfaces revealed a significant decrease of the percentage of Ki67^+^ tissue for combination **I** only (37% inhibition vs. CTRL; *P < 0.05; Fig. 5E). Representative images of treated tumors show a decrease of tissue with viable proliferating cells (Fig. 5G). Since cisplatin is the first-line treatment for patients with high-stage ovarian carcinoma, the *in vivo* activity of erlotinib/RAPTA-C combinations was also determined on A2780 tumors with an induced resistance to cisplatin. This might demonstrate a therapeutic potential in the treatment of patients with ovarian carcinoma showing resistance to standard therapy. We hypothesized, based on the *in vitro* data, that erlotinib/RAPTA-C combination treatment may efficiently inhibit tumor growth in the A2780cisR ovarian carcinoma tumors as the drug combination appears to operate via a different mechanism than that of cisplatin. Similar to the tumors developed from treatment naïve A2780 cells, single drug administration only slightly reduced A2780cisR tumor growth (Fig. 6A). Treatment with combination **I** resulted in 58% inhibition of tumor growth (**P < 0.01 vs. CTRL and single drug treatments; CI = 0.07) on the last treatment day. For combination **II,** tumor growth inhibition of 67% was observed (**P < 0.01 vs. CTRL; CI = 0.69). Notably, embryo weight was assessed on the last day of the experiment and was not significantly affected by any combination treatment in either tumor model (Supplementary Figure 8), suggesting a lack of toxicity of the combination treatments.

Interestingly, while combination **II** treated tumors showed similar efficacy in A2780 and A2780cisR (64% vs. 67% tumor growth inhibition respectively), combination **I** is more effective in A2780cisR tumors as compared to the non-resistant variant (34% vs. 58% tumor growth inhibition for A2780 and A2780cisR tumors, respectively). This difference in activity is supported by the response surfaces shown in Fig. 2. In particular, a clear lack of activity of combination **I** can be seen in the A2780 cell line while its activity in the A2780cisR cell line is relatively strong.

Quantification of CD31 stained A2780cisR tumor sections revealed a decrease in MVD for erlotinib and RAPTA-C mono-therapies (i.e. 19% for erlotinib at 20 μg/kg/day and 28% for RAPTA-C at 216 μg/kg/day, Fig. 6C). In both cases, tumor growth was also reduced, although not significantly (Fig. 6A). Erlotinib/RAPTA-C combination treatments at both dose-ratios resulted in a decrease in MVD, although this decrease was only significant for combination **II** (34% inhibition; vs. CTRL; **P < 0.01; Fig. 6C). Quantification of IHC samples stained for the proliferation marker Ki67 showed a significant decrease in the percentage of Ki67^+^ tissue for combination **I** only (16% inhibition vs. CTRL; **P < 0.01; Fig. 6D).

### Inhibition of tumor growth in a murine xenograft model by erlotinib/RAPTA-C combinations

**Figure 7:**
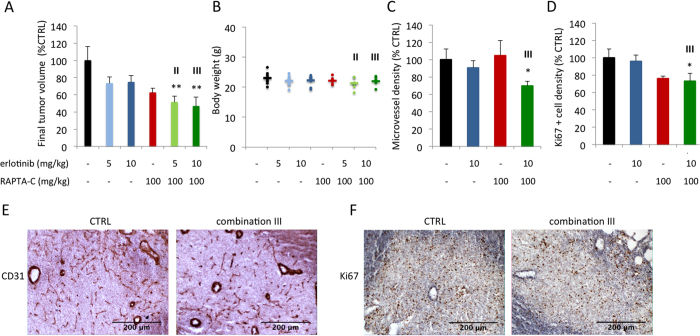
Inhibition of A2780 tumor growth in nude mice by erlotinib, RAPTA-C and their combination treatment. (**A**) Resected tumor volume of each treatment group as the percentage control on treatment day 12. N = 5–7 in all treatment groups. Control tumors were treated i.p. with 4% DMSO in NaCl (One-way ANOVA with Tukey’s multiple comparisons test: F(5,33) = 4.145, P = 0.0050). Pharmacokinetic studies in mice revealed a plasma half-life time of 1.5–3 hours after ingestion for erlotinib and 10.39–12.21 hours after ingestion for RAPTA-C^16^,^69^. (**B**) Body weight (g) measured at treatment day 12. (**C**) Microvessel density (MVD) analysis in combination **III** treated tumors represented as the number of vessels per mm^2^ of vascularized tumor area and represented as percentage of control. (**D**) Analysis of staining for the proliferation marker Ki67 in combination **III** treated tumors shown as the density of Ki67 positive nuclei per image field and represented as percentage CTRL. (**E**) Representative images of IHC staining for the endothelial cell marker CD31 (brown) counter-stained with haematoxylin (purple/blue). (**F**) Representative images of IHC staining for Ki67 (brown/orange) counter-stained with haematoxylin (purple/blue). **III** indicates the combination erlotinib 10 mg/kg/day + RAPTA-C 100 mg/kg/day in all graphs. Error bars represent SEM and *P < 0.05 indicates significance vs. CTRL. Statistical analysis was performed using a one-way ANOVA with post-hoc Tukey’s multiple comparison test (**A**), or an unpaired, one-tailed t-test with Welch’s correction.

The anti-tumor activity of erlotinib/RAPTA-C combinations (erlotinib at 5 mg/kg/day and RAPTA-C at 100 mg/kg/day; referred to as combination **II**; erlotinib at 10 mg/kg/day and RAPTA-C at 100 mg/kg/day; referred to as combination **III**) was further investigated in nude mice xenografted with A2780 tumors. Treatment with combination **II** and **III** resulted in similar levels of additive tumor growth inhibition (48 and 53%, respectively; **P < 0.01 vs. CTRL; Fig. 7A), whereas treatment with erlotinib 5 mg/kg and 10 mg/kg, or RAPTA-C (100 mg/kg) as mono-therapies inhibited tumor growth by only 27%, 25%, and 37%, respectively. Notably, the average body weight of the mice measured on the last day of the experiment was not significantly affected by any of the treatments, indicative of a lack of drug toxicity (Fig. 7B). Assessment of staining for CD31 in tumor sections revealed that the combination treatment significantly decreases MVD (by 30%), whereas the mono-therapies had little effect (*P < 0.05; Fig. 7C). Furthermore, assessment of the density of Ki67^+^ nuclei in tumor sections revealed a decrease in the density of proliferating cells (27% inhibition; *P < 0.05; Fig. 7D). These results indicate that the erlotinib/RAPTA-C combination reduces tumor growth via both antiangiogenic and anti-proliferative effects.

## Discussion

An *in vitro* high-throughput screening-study revealed potential synergistic interactions between the EGFR inhibitor erlotinib and the broad acting organometallic compound RAPTA-C. In the present study, we performed an in-depth investigation of this drug combination and demonstrate their strong synergistic antitumor efficacy in preclinical tumor models, including one drug-resistant tumor model.

Previous studies have shown anti-angiogenic and anti-tumor effects after treatment with erlotinib and cisplatin. Combinations of these two drugs showed enhanced anti-angiogenic activity in non-small cell lung cancer (NSCLC) cells due to decreased VEGF levels and was evidenced by a decrease in the vessel density of NSCLC tumor xenograft samples^50^. We have previously demonstrated that RAPTA-C and other related Ru(II) compounds exhibit anti-angiogenic activity^23^. Furthermore, the Ru(III) complex NAMI-A has been shown to induce apoptosis in the spontaneously transformed human endothelial cell line, ECV304^51^. Synergistic interactions have previously been reported between KP1339 and the multi-kinase inhibitor sorafenib in hepatoma cancer both *in vitro* and *in vivo*^26^. Moreover, our previous screen did not show synergy in ECs when sorafenib, sunitinib and the mTOR inhibitor BEZ-235 were tested in combination with RAPTA-C at various dose ratios in our experimental setting^34^.

The analysis of the cellular uptake of ruthenium via inductively coupled plasma mass spectrometry (ICP-MS) revealed that the co-administration of erlotinib with RAPTA-C significantly enhances the uptake of Ru in both A2780 and A2780cisR cancer cell lines as compared to the Ru uptake of RAPTA-C administration alone (Fig. 2B). These results concur with previous studies which identified increased cellular uptake of Ru as one of the mechanisms leading to synergistic interactions between KP1339 and sorafenib^26^.

Our mechanistic analysis suggests two pathways by which the combination of erlotinib and RAPTA-C impair the growth of different cell types. While significant apoptosis induction was seen in endothelial ECRF24 and HUVEC cells, ovarian cancer A2780 and A2780cisR cells showed DNA bridges that prevent cytokinesis. Future investigation, possibly by live cell imaging, will have to uncover the precise origin of these DNA bridges. Nevertheless, since cytokinesis failure leads to a p53-dependent cell cycle arrest (via the hippo pathway) and cell senescence^52^,^53^, we postulate that growth impairment seen in A2780 and A2780cisR is due to senescence, consistent with the lack of apoptotic cells.

The investigation of the anti-tumoral activity of varying dose ratios of combinations of erlotinib and RAPTA-C revealed effective inhibition of tumor growth in both A2780 and A2780cisR tumors grown in the CAM model. Differences in response between cell lines and dose ratios indicate that drug dose ratios can dramatically impact the overall efficacy of a drug combination and, more importantly, that higher drug doses do not necessarily correlate to higher efficacies, as observed in the *in vitro* assays. These results suggest that by using optimal drug dose ratios it is possible to target both the endothelial and tumor cells more efficiently. In this case, the inhibition of MVD limits tumor growth to a certain extent, however, part of the mechanism of drug action is also through the direct targeting of tumor cells. The microvessel density (MVD) in treated A2780 tumors, quantified by staining for the endothelial cell marker CD31, revealed that treatment with combinations of erlotinib and RAPTA-C decreased MVD by 37% (combination **I**; **P < 0.01; Fig. 5D) and by 34% (combination **II**; *P < 0.05; Fig. 5D). We have previously described the activity of RAPTA-C as single drug therapy in the same model when administered at 200 μg/kg/day for 5 consecutive days. A decrease in MVD of approximately 80% was observed which corresponded to a tumor growth inhibition of 82%^22^, however, a longer treatment duration was required compared to when RAPTA-C is administered in combination with erlotinib. Moreover, succesful translation of anti-tumor efficacy to a nude mice A2780 xenograft model was achieved since treatment with erlotinib/RAPTA-C resulted in significant tumor growth inhibition, reduced MVD, and a reduction in proliferating tumor cells (Fig. 7).

**Figure 8:**
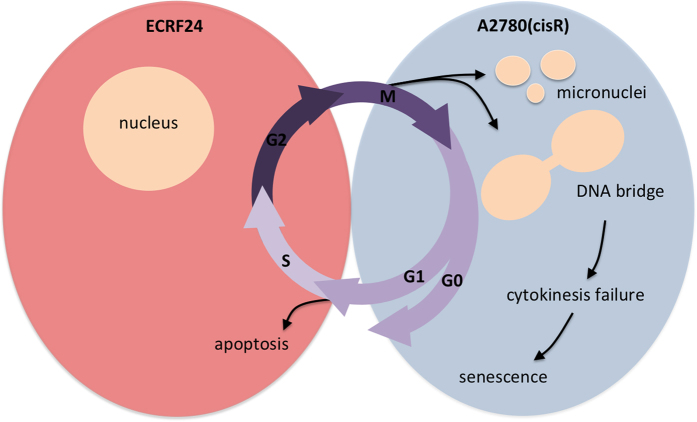
Schematic representation of cellular mechanism involved in the activity of erlotinib and RAPTA-C combinations. In ECRF24 cells, DNA damage may lead to failure to progress through G1/S checkpoint and therefore induction of apoptosis. In A2780 and A2780cisR cells mitotic defects lead to the formation of DNA bridges and micronuclei. Via the formation of DNA bridges, cytokinesis might be blocked leading to senescence.

Taking into account the *in vitro* results of combination **II** in both endothelial and cancer cell types (Figs 1 and 3, i.e. combination **II** treatment inhibits viability in both cell types, while enhancing apoptosis induction in endothelial cells, and increasing ruthenium uptake together with significantly increasing the percentage of cells with DNA bridges or micronuclei in tumor cells), the *in vivo* activity seen in all *in vivo* models results from varied mechanisms of actions in tumor cells and their microenvironment (Fig. 8).

In the *in vivo* models tested, MVD decreased significantly following treatment with the combination therapy whereas endothelial cell mobility was not affected (Supplementary Figure 1B), which is consistent with previous studies^34^. Supporting evidence suggests that inhibition of cell mobility alone does not have a therapeutic effect whereas the inhibition of endothelial cell viability alone does^54^. This evidence, together with the data presented here, indicates that inhibition of cell viability may be more dominant in accounting for an angiostatic anti-tumor effect as compared to inhibition of cell mobility.

In conclusion, erlotinib and RAPTA-C combination therapy synergistically inhibits endothelial and ovarian carcinoma cell proliferation, but does not affect cell mobility. Furthermore, the drug combination was shown to target angiogenesis in *in vivo* bioassays and in tumor growth inhibition models. RAPTA-C was previously shown to exhibit anti-metastatic activity, as well as having activity against primary tumors, to inhibit angiogenesis in various models and to have a direct cytotoxic effect on tumor cells while showing little toxicity^16^,^22^,^23^,^34^. Consequently, RAPTA-C may serve as a good alternative to platinum-based drugs in combination therapies, especially in ovarian carcinoma, where increased activity of EGFR targeting drugs have been observed when combined with cytotoxic agents^55^,^56^.

## Methods

### Compounds

Erlotinib was purchased from LC laboratories (Woburn, MA, USA) and was dissolved in sterile DMSO at a concentration of 15 mg/ml. Aliquots were stored in −80 °C and thawed prior to each experiment. RAPTA-C (Ru(η^6^-*p*-cymene)Cl_2_(pta)], (pta = 1,3,5-triaza-7-phosphaadamantane) was synthesized and purified as described previously^22^. RAPTA-C powder was freshly dissolved in DMSO at 100 mg/ml before each experiment. The maximum DMSO percentage was 0.14% and showed minimal activity in the performed assays.

### Cell cultures

Immortalized human vascular endothelial cells (ECRF24) were cultured in medium containing 50% DMEM and 50% RPMI-1640 medium supplemented with 10% fetal bovine serum (FBS) and 1% penicillin/streptomycin (Life Technologies, Carlsbad, California, USA). Human ovarian carcinoma cells were cultured in DMEM (A2780) and RPMI-1640 (A2780cisR) supplemented as above. HEK-293 cells were cultured in DMEM and supplemented as above. Human umbilical vein endothelial cells (HUVEC) were isolated from human umbilical cords as previously described and cultured in 0.22 μm filtered RPMI-1640 medium supplemented with 10% human serum (HS), 10% FBS, 1% penicillin/streptomycin and 2 mM L-glutamine (Life Technologies)^57^. PBMCs were freshly isolated as previously described and cultured in IMDM supplemented with 10% FBS, 1% penicillin/streptomycin and 0.05 β-mercaptoethanol^58^. ECRF24 and HUVEC were always cultured on 0.2% gelatin coated surfaces. All cell lines were maintained in a humidified incubator (5% CO_2_) at 37 °C.

### Cell viability, cell migration, and apoptosis

For cell viability experiments, cells were seeded in the inner 60 wells of a 96-well culture plate at a density of 2.5–10 × 10^3^ cells/well (depending on cell type) and grown for 24 h. Cells were allowed to grow for an additional 72 h in the presence of medium or drugs after which cell viability was assessed with the CellTiter-Glo luminescence assay (Promega, Madison, WI, USA). Cell response to drug treatment was determined based on normalizing the luminescence signal in the treated wells as compared to medium controls.

Cell migration was assessed with the wound healing assay as previously described^59^. ECRF24 cells were seeded at a density of 30 × 10^3^ cells/well in a 96-well culture plate and grown overnight to confluence. A scratch wound was made on the confluent cell layer with a sterile scratch tool (Peira Scientific Instruments, Beerse, Belgium) and drugs or medium was applied to the cells. Scratch wound surfaces were automatically imaged with a Leica DMI3000 microscope (Leica, Rijswijk, Netherlands) at 5x magnification using Universal Grab 6.3 software (DCILabs, Keerbergen, Belgium). Images of scratch wounds were taken at T = 0 h and T = 6 h and scratch surfaces were analyzed by Scratch Assay 6.2 (DCILabs). Cell migration was quantified by calculating the absolute wound closure (initial minus final scratch surface) and values were presented as the percentage of medium treated control wound closure.

Flow cytometry analysis was performed to identify cell cycle stages including apoptotic fractions. Cells were seeded in 24-well plates at a density of 20–40 × 10^3^ cells/well and incubated for 24 h. Medium or drugs were applied and cells were incubated for an additional 72 h. Cells were harvested by trypsinization, suspended in 70% ethanol and incubated for 2 h at −20 °C. Cell pellets were then re-suspended in DNA extraction buffer containing 90 parts 0.05 M Na_2_HPO4, 10 parts 0.025 M citric acid, 1 part 10% Triton-X100, (pH 7.4) and incubated for 20 min at 37 °C. Propidium iodide (PI, 20 μg/ml) was added and cells were analyzed with a FACSCalibur flow cytometer (BD Biosciences) in the FL2 channel. Apoptotic cells were defined as having subG1 DNA staining and quantified with CellQuest Pro software (BD Biosciences). For cell growth monitoring, A2780 and A2780cisR cells were seeded at 10000 c/well in a 96-welll plate and allowed to attach overnight followed by treatment of the cells with the following conditions: DMSO control, 5 μM erlotinib, 10 μM erlotinib, 10 μM RAPTA-C, 100 μM RAPTA-C, combination I and combination II. Photographs of wells were taken using LEICA DMI 3000B before treatment, then at 24 h, 48 h and 72 h after treatment. To quantify cell density, Scratch Assay software (DCI labs) was used, which reports cell covered areas (μm^2^).

### Stress fibre formation assay

A2780 and A2780cisR cells were seeded in a 24-well plate and allowed to adhere overnight. Cells were then pretreated for 24 h with the following conditions: DMSO control, 5 μM erlotinib, 10 μM erlotinib, 10 μM RAPTA-C, 100 μM RAPTA-C, combination I and combination II. Cells were washed with PBS, then fixed with 1% paraformaldehyde (Electron Microscopy Sciences-Aurion, Hatfield, PA) for 20 min and consequently permeabilized with 0.1% Triton x-100 (Amresco, Solon, Ohio) for 10 min at 37 °C. Cells were incubated with Rhodamine-Phalloidin (Invitrogen, Oregon, USA) and DAPI (Invitrogen, Oregon, USA) in warmed 0.1% bovine serum albumin for 40 min. Cells were observed and photographed using a fluorescence microscope (LEICA DFC 345FX).

### Immunoblotting

A2780 cells were seeded in 6-well plates and allowed to adhere overnight. Cells were pretreated either with DMSO control, 5 μM erlotinib, 100 μM RAPTA-C or combination of 5 μM erlotinib and 100uM RAPTA-C for 2 h at 37 °C. Cells were lysed in radioimmunoprecipitation assay buffer (25 mM Tris (pH 7–8), 150 mM NaCl, 0.1% SDS, 0.5% sodium deoxycholate, 1% Triton-X-100) supplemented with Halt^TM^ Protease/Phosphatase inhibitor cocktail (Thermo Scientific, Rockford, USA) on ice and collected. Cell lysate was vortexed and snapfrozen in liquid nitrogen followed by sonication at 20 Amp for 10 s on 10 s off. Supernatant was collected by centrifugation at 15,000 *g* at 4 °C for 10 minutes. Protein concentration was determined using the Micro BCA™ Protein Assay Kit (Thermofisher Scientific, Rockford, USA). Equal amounts of protein were resolved on SDS-PAGE and transferred to a nitrocellulose membrane. The membrane was blocked with 5% bovine serum albumin in TBS-T and incubated with primary antibodies against total and phospho- FAK, AKT (Cell Signalling, Danvers, MA) and ERK (New England, Biolabs, Ipswich, MA) at 4 °C overnight with constant rolling. The housekeeping protein GAPDH was used as internal control (Cell Signalling, Danvers, MA). The membrane was washed with TBS-T 3 × 20 min, incubated with IRdye^®^ 680RD goat anti-rabbit secondary antibody (LICOR, Westburg, EU) for 30 min at room temperature with constant rolling and detected by the Odyssey^®^ infrared imaging system.

### Cellular uptake of Ruthenium-102

Cells were seeded in 6-well cell culture plates with either 5 × 10^5^ (ECRF24 and A2780cisR) or 3 × 10^5^ (A2780) cells per well and allowed to adhere overnight. Subsequently, cells were treated with either medium (CTRL), single drug treatment of RAPTA-C or the combination of erlotinib/RAPTA-C and incubated for 5 h. Cells were then washed 3 times with ice cold phosphate-buffered saline (PBS) and detached using PBS-Based Enzyme Free-Cell Dissociation Solution (Millipore™ Specialty Medium, Cat. # S-014-B). Next, cell lysis was achieved using the ‘freeze-thaw’ method, i.e. by performing 5 cycles of cooling the cells at −20 °C for 20 min and warming the cells at +37 °C for 20 min. The cell lysate was then stored at −20 °C until the day of the analysis.

Ten μl of each sample was isolated for protein quantification using the Quick Start™ Bradford Protein Assay based on the manufacturer’s instructions. The samples were subsequently digested for 3 h at 37 °C by adding 150 μl of 65% nitric acid. Following digestion, each sample was transferred to a 15 mL tube and diluted with water containing (1 ppb indium as an internal standard) to a final solution containing 3% nitric acid. ICP-MS was performed using an Elan DRC II ICP-MS instrument (Perkin Elmer, Switzerland) equipped with a Meinhard nebulizer and a cyclonic spray chamber.

### Photodynamic therapy (PDT)-induced vessel sprouting on the chicken chorioallantoic membrane (CAM)

The experiments were carried out in accordance with relevant guidelines and regulations. The use of the CAM model in the Netherlands does not require the approval of the experimental protocol. Visualization of the CAM vasculature and irradiation with light during PDT was performed under an epi-fluorescence microscope (Eclipse E 600 FN; Nikon AG, Tokyo, Japan) with objectives (Plan Apo 4 ×/0.2, working distance: 20 mm or Plan Fluor 10 ×/0.3, working distance: 16 mm; Nikon AG), as previously described^60^. Shortly, PDT was performed using Visudyne^®^ (Novartis Pharma Inc., Hettlingen, Switzerland) with a 5 J/cm^2^ light energy dose at an irradiance of 35 mW/cm^2^ (λ_ex_ = 420 ± 20 nm, λ_em_ ≥ 470 nm) on EDD11. The irradiation area was limited to a circular spot of 0.02 cm^2^ using an optical diaphragm. Directly after PDT, 20 μl of drugs or drug combinations were administered intravenously. Visualization of blood vessels was performed 24 h after treatment (EDD12) through fluorescence angiography after intravenous (i.v.) injection of fluorescein isothiocyanate dextran (FITC-dextran, 20 kD, 20 μl, 25 mg/ml, Sigma-Aldrich). A volume of 20 μl of India ink from Pelikan (Witzikon, Switzerland) was administered in the embryonic cavity to enhance vascular contrast. Fluorescence images were taken using a pco.1300 12-bit CCD camera (Gloor Instruments AG, Usler CH) run by Micro-Manager 1.4 (National Institutes of Health, Bethesda, MD, USA)^61^. Image processing and quantification of the fluorescence angiographies was achieved by using a macro written in ImageJ (version 1.40 a; National Institutes of Health, Bethesda, MD, USA), as previously described^62^. The parameters number of sprouts, average segment length and branching points per mm^2^ were quantified. Effects on the mature vasculature in the CAM were assessed through FITC-dextran visualization of the surrounding (non-PDT treated) vasculature 24 h after i.v. drug administration.

### Human ovarian carcinoma grown on the chicken chorioallantoic membrane (CAM)

Fertilized chicken eggs were incubated in a hatching incubator (relative humidity 65%, 37 °C), as previously described^63^. On embryo development day (EDD) 8, A2780 or A2780cisR cells were prepared in 25 μL hanging spheroids containing 10^6^ cells in 20% Methocel (Sigma-Aldrich) and 80% serum free RPMI-1640 medium. The spheroids were then incubated for 3 h and transplanted on the surface of the CAM. Vascularized three-dimensional tumors were visible 3 days after tumor cell implantation and randomized on EDD 11, when treatment was initiated. Tumors were treated on EDD 11 and 12 (referred to as treatment day 1 and 2) by 20 μl intravenous injection into a main blood vessel of the CAM. Erlotinib and RAPTA-C were freshly prepared and pre-mixed in 0.9% NaCl. The injected doses (10–20 μg/kg for erlotinib and 21.6–216 μg/kg for RAPTA-C) were adjusted to the normalized embryo weight at EDD 11/12 (4.27 g). Control tumors were treated with vehicle (0.14% DMSO in 0.9% NaCl). Tumors were monitored daily for 8 days and tumor size was calculated with the formula: volume = [large diameter] x [perpendicular diameter] 2 × 0.52. At EDD 18: (i) the embryos were sacrificed and weighed and (ii) the tumors were resected, weighed and fixed in zinc-fixative for additional analysis.

### Human ovarian carcinoma xenograft model

All methods were carried out in accordance with relevant guidelines and regulations and experimental protocol was approved by the Committee for Animal Experiments for the Canton Vaud, Switzerland. Female Swiss nu/nu mice ages 6–8 weeks were obtained from Charles River (weight 20–30 grams). Mice were inoculated in the right flank with 100 μl RPMI containing 5 million A2780 cells. Tumors treatment was initiated 6 days after inoculation, when palpable tumors had appeared. Mice were treated daily by oral gavage with erlotinib 10 mg/kg (suspended in sterile water with 0.5% (w/v) methyl cellulose and 0.1% (v/v) tween 80), by i.p. injection with RAPTA-C 100 mg/kg (freshly dissolved in DMSO and diluted in 0.9% NaCl) both in a total volume of 100 μl or the combination of both treatments. The dose of RAPTA-C was selected based on previous experiments of RAPTA-C in a murine xenograft model (LS174T) where erlotinib 5 mg/kg/day and RAPTA-C 40 mg/kg/day showed synergy but was minimally effective^34^. Therefore, in this study, the dose of RAPTA-C was increased to 100 mg/kg/day. Moreover, the erlotinib dose of 10 mg/kg/day was selected as a reduced dose to show 10–40% anti-tumour efficacy, as seen in *in vitro* assays, and being well below the reported MTD of 100 mg/kg/day in mice^64^. Mice in the control group were treated by i.p. injection of 0.9% NaCl containing 4% DMSO, to mimic the solution of RAPTA-C. Mice were monitored daily for tumor size and body weight.

### Immunohistochemistry

Immunohistochemical (IHC) staining of CAM tumors with endothelial cell marker CD31, to detect blood vessels, and with proliferation marker (in cell nuclei) Ki67 was performed as previously described^65^. Briefly, tumors were fixed overnight in zinc fixative solution, embedded in paraffin and 5 *μ*m sections were stained. Sections were blocked with 5% bovine serum albumin (BSA) in PBS followed by incubation with primary antibodies against CD31 (1:200; clone SZ31, Dianova, Hamburg, Germany). Secondary donkey anti-rat biotinylated antibodies (1:200; Jackson, Suffolk, UK) were then incubated, followed by streptavidin-HRP (1:50; Dako, Glostrup, Denmark) and visualized by 3,3′-diaminobenzidine (DAB), resulting in a brown-colored precipitate at the antigen site. Sections were stained separately for proliferation marker with primary antibodies against Ki67 (rabbit anti-human Ki67, 1:100, clone SP6; Thermo Scientific) and subsequent incubation with secondary swine anti-rabbit biotinylated antibodies (1:200; Dako, Glostrup, Denmark). Ki67^+^ nuclei were visualized by DAB following streptavidin-HRP staining. Microvessel density (MVD) in CAM and mouse tumors was assessed by ImageJ quantification of representative images (20x objective) within vascular hotspots using the colour deconvolution plugin, as previously described^66^. When necessary, vessel counts were corrected for the presence of necrotic patches or holes in an image. In mouse tumors, the density of Ki67 positive nuclei per image field (20x objective) was also assessed by the colour deconvolution in ImageJ. In CAM tumors, the percentage of Ki67 positive tumor areas was calculated by measuring images (4x objective) of the total tumor area and areas with Ki67 staining in ImageJ.

### Statistical analysis

The data is presented as the mean of multiple independent experiments (±SEM). Significance was determined using the one-way or two-way ANOVA test with post-hoc Tukey’s multiple comparison test, or post-hoc Dunnett’s multiple comparison test between the combination and control and an unpaired t test with Welch’s correction (Graphpad Prism). *P values lower than 0.05 and **P lower than 0.01 were considered statistically significant and are indicated versus the control in all figures. Combination index (CI) values were determined by the Chou-Talalay method using the Compusyn software, as previously described^67^,^68^. When the single drug efficacy of a compound was above 100% (i.e. stimulatory), a value of 0.001 was used for the fraction affected in order to calculate the CI values. In the case of A2780cisR, which was not responsive to RAPTA-C, artificial values of efficacy were used with a maximal effect of 2% for the highest dose of 200 μM.

## Additional Information

**How to cite this article**: Berndsen, R. H. *et al*. Combination of ruthenium(II)-arene complex [Ru(η6-*p*-cymene)Cl_2_(pta)] (RAPTA-C) and the epidermal growth factor receptor inhibitor erlotinib results in efficient angiostatic and antitumor activity. *Sci. Rep.* **7**, 43005; doi: 10.1038/srep43005 (2017).

**Publisher's note:** Springer Nature remains neutral with regard to jurisdictional claims in published maps and institutional affiliations.

## Supplementary Material

Supplementary Information
