# Supplementary material for: Combination of ruthenium(II)-arene complex [Ru(η6-p-cymene)Cl2(pta)] (RAPTA-C) and the epidermal growth factor receptor inhibitor erlotinib results in efficient angiostatic and antitumor activity
Source: Sci Rep. 2017 Feb 22;7:43005. doi: 10.1038/srep43005 (PMC5320450; doi:10.1038/srep43005)
Supplement: Supplementary Information [file srep43005-s1.pdf]

Supplementary information:

Combination of ruthenium(II)-arene complex [Ru(η^6-*p*-cymene)Cl_2(pta)] (RAPTA-C) and the epidermal growth factor receptor inhibitor erlotinib results in efficient angiostatic and antitumor activity

Robert H. Berndsen[1], Andrea Weiss[2], U. Kulsoom Abdul[1], Tse J. Wong[1], Patrick Meraldi[3], Arjan W. Griffioen[1], Paul J. Dyson[2] and Patrycja Nowak-Sliwinska[4]*

[1]Angiogenesis Laboratory, Department of Medical Oncology, VU University Medical Center, Amsterdam, The Netherlands, [2]Institute of Chemical Sciences and Engineering, Swiss Federal Institute of Technology (EPFL), Lausanne, Switzerland, [3]Department of Cell Physiology and Metabolism, University of Geneva Medical School, University of Geneva (UNIGE), Geneva, Switzerland, [4]School of Pharmaceutical Sciences, University of Geneva (UNIGE), Geneva, Switzerland

[*] Correspondence to:
Patrycja Nowak-Sliwinska, School of Pharmaceutical Sciences, University of Geneva (UNIGE), Rue Michel-Servet 1, CMU6 - A06.2914, 1211 Geneva 4, Switzerland, Tel: +41 22 379 3352, Fax: +41 22 379 6089, e-mail: Patrycja.Nowak-Sliwinska@unige.ch

Contents:

Supplementary information
Supplementary table
Supplementary figures

Supplementary information:

Autophagy assay

The autophagy assay was performed by flow cytometry analysis of autophagic vacuoles labeled with the fluorescent compound monodansylcadavarine (MDC). Cells were seeded in 24-well plates at a density of 100 x 10^3 cells/well and incubated overnight. Cells were harvested by trypsinization, washed and incubated with 0.05 mM MDC at 37°C for 1 h. Cells were then washed and analyzed directly with a FACSCalibur flow cytometer (BD Biosciences) in the FL1 channel. Bafilomycin 100 nM (Sigma – Aldrich) served as negative control..

Supplementary table:

Table 1. Cell type specific combination index (CI) for cell viability inhibition.

	erlotinib (µM) / RAPTA-C (µM)									
	1/10[a]	5/10	10/10	5/50	10/50	5/100	10/100	5/200	10/200	15/200
**RF24**	0.05[b]	0.13	0.21	0.12	0.21	0.10	0.22	0.11	0.17	0.20
**HUVEC**	0.50	0.65	0.85	0.81	0.77	0.51	0.80	0.80	0.65	0.73
**A2780**	0.05	0.11	0.65	0.26	1.36	0.19	0.16	0.33	0.34	0.31
**A2780cisR**	0.05	1.19	0.29	0.35	0.30	0.44	0.92	0.64	0.99	1.91

Table 1. [a]Represents the concentration of erlotinib 1µM and RAPTA-C at 10 µM. [b]CI values were calculated with CompuSyn software based on the cell viability inhibition[55]. CI <1 indicates a synergistic effect, CI=1 an additive effect and CI>1 indicates antagonism.

Supplementary figures

Supplementary Figure 1

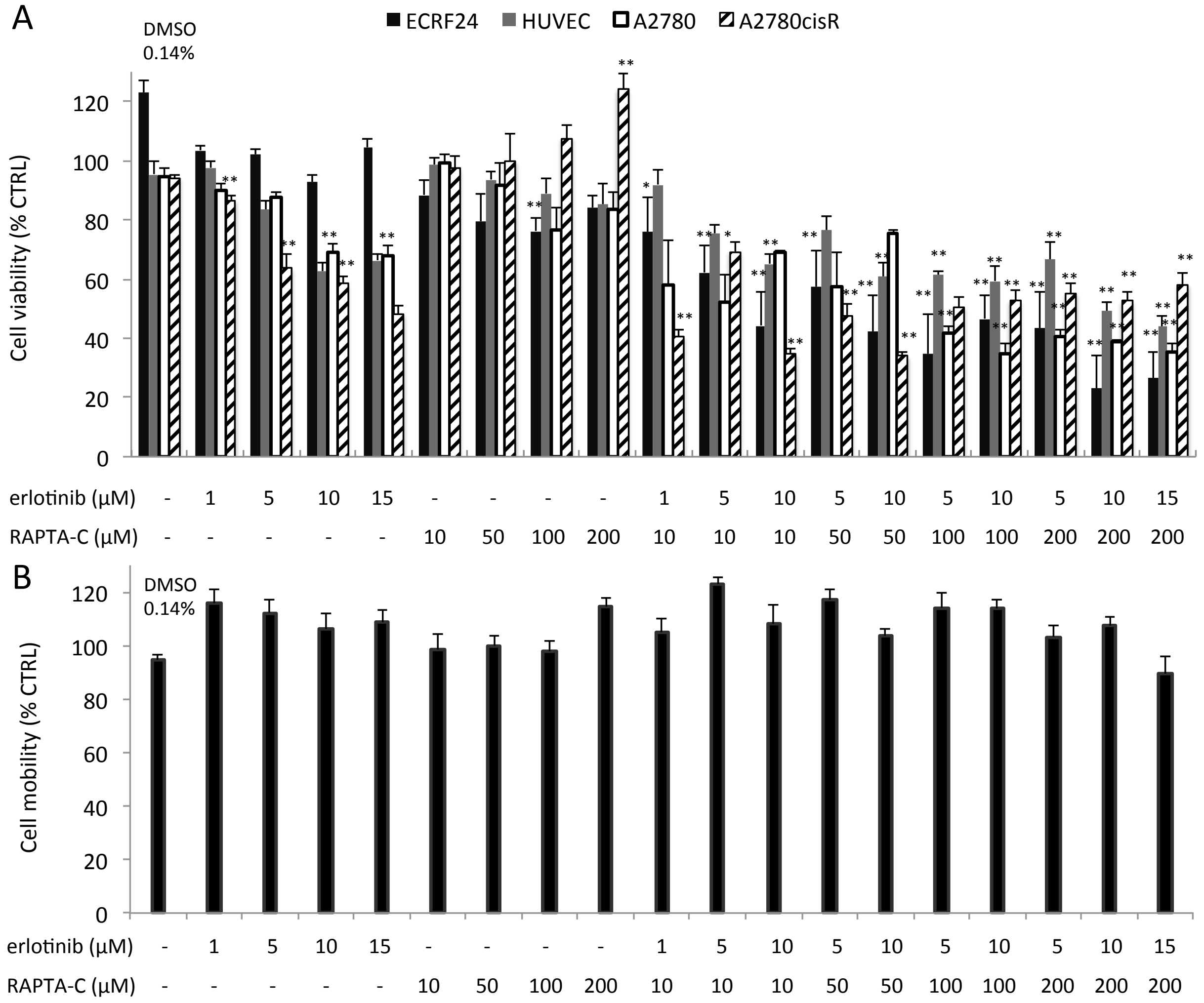

Supplementary Figure 1. Activity of erlotinib and RAPTA-C on the inhibition of endothelial and ovarian cancer cell viability (A) and the migration on ECRF24 cells (B). (A) Inhibition of cell viability by erlotinib, RAPTA-C and their combinations in endothelial cells (immortalized ECRF24 and primary human umbilical vein endothelial cells; HUVEC) and ovarian cancer cells (A2780 and A2780cisR). Cell viability was assessed after 72 h of exposure by the CellTiter-Glo luminescence assay and represented as a percentage of the control. Significance is indicated vs. CTRL (0.14% DMSO treated cells), with * $P<0.05$ and ** $P<0.01$, based on a one-way ANOVA with post-hoc Tukey's test (ECRF24: $F(19, 219) = 13.69$, $P<0.0001$; HUVEC: $F(19, 175) = 16.83$, $P<0.0001$; A2780: $F(19, 85) = 10.86$, $P<0.0001$; A2780cisR: $F(19, 205) = 58.2$,

P<0.0001). (B) Cell mobility inhibition induced by erlotinib, RAPTA-C and their combinations in the ECRF24 cell line. Cell mobility was assessed 6 h after a scratch wound was made in a cell monolayer. Values represent the mean of at least two independent experiments performed in triplicate and error bars represent the SEM.

Supplementary Figure 2

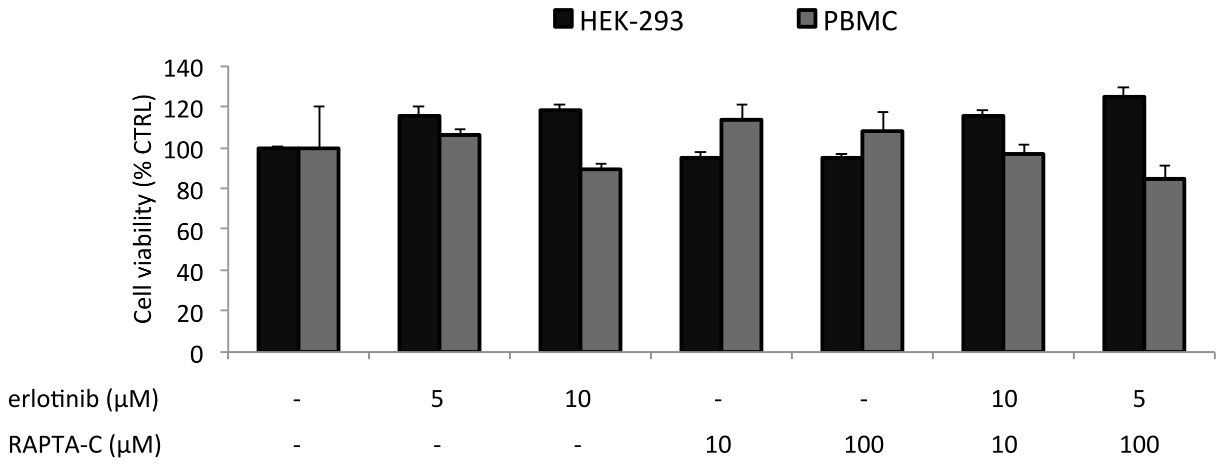

Supplementary Figure 2. Activity of erlotinib and RAPTA-C on the inhibition of human embryonic kidney cells (HEK-293) and peripheral blood mononuclear cell (PBMC) viability. Cell viability was assessed after 72 h of exposure by the CellTiter-Glo luminescence assay and represented as a percentage of the control. Statistical analysis was performed by one-way ANOVA with post-hoc Tukey's test (HEK-293T: $F(3, 11) = 20.07$, $P<0.0001$ and PBMC: $F(3, 20) = 0.7879$, $P=0.5148$;). Values represent the mean of at least two independent experiments performed in triplicate and error bars represent the SEM.

Supplementary Figure 3

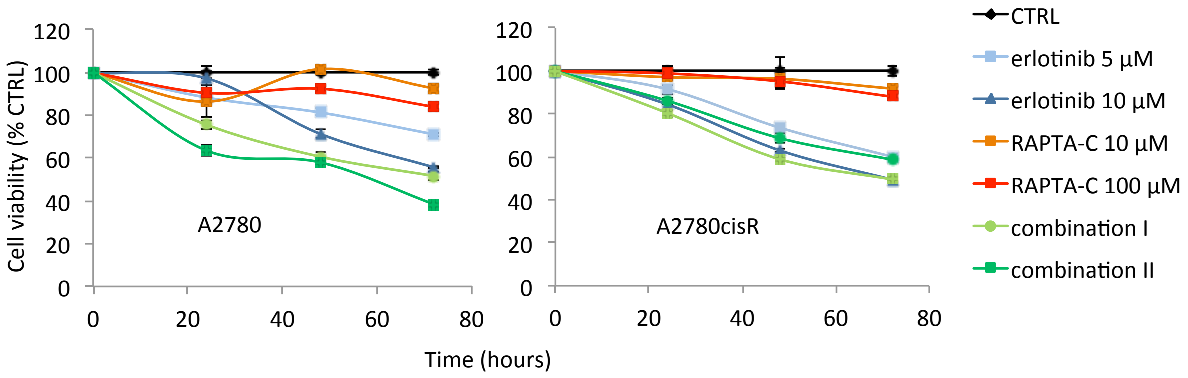

Supplementary Figure 3. Inhibition of cell viability by erlotinib, RAPTA-C and their combinations in ovarian cancer cells (A2780 and A2780cisR). Combination I represents erlotinib 10 µM/RAPTA-C 10 µM and combination II represents erlotinib 5 µM/RAPTA-C 100 µM. Cell viability was assessed after 24, 48 and 72 hours of treatment. Cell viability was determined by the CellTiter-Glo luminescence assay and represented as a percentage of the control. Values represent the mean of at least two independent experiments performed in triplicate and error bars represent the SEM.

Supplementary Figure 4

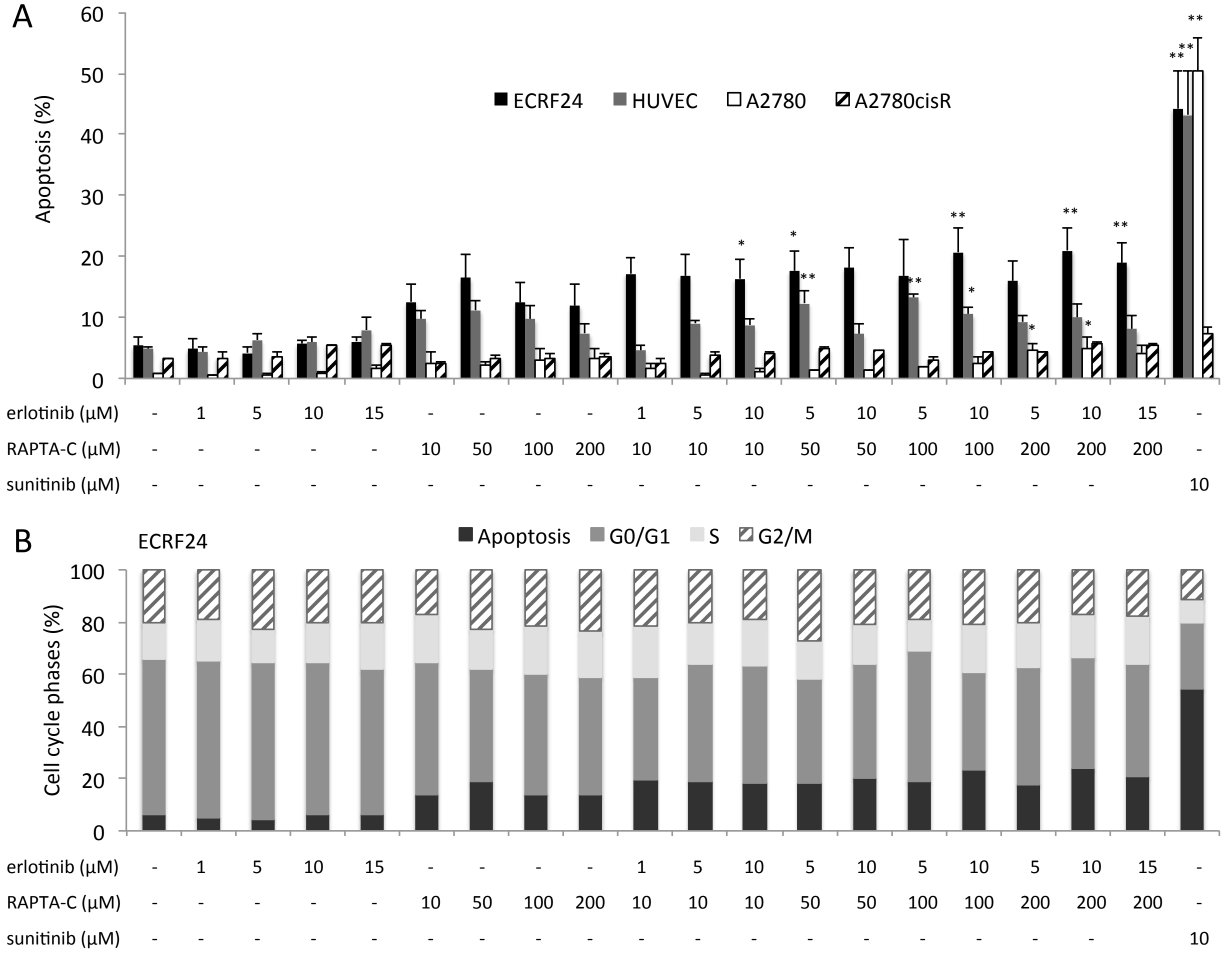

Supplementary Figure 4 . Apoptosis induction and cell cycle distribution in human endothelial and ovarian carcinoma cells. Apoptosis and cell cycle distributions were assessed using flow cytometry analysis based on propidium iodide (PI) staining of DNA. Sunitinib (10 µM) was used as a positive control. (A) Apoptosis induction of erlotinib, RAPTA-C and ten different combinations in ECRF24, HUVEC, A2780 and A2780cisR cells. (B) Cell cycle distribution in ECRF24. Values represent the mean of 2-5 independent experiments and error bars represent SEM. Statistical analysis was performed based on a one-way ANOVA with post-hoc Dunnett's multiple comparison test performed between the combination and control groups only (ECRF24: $F(10,42)=2.125$, $P=0.0421$; HUVEC: $F(10,29)= 3.369$, $P=0.0051$; A2780: $F(10,21)=3.492$, $P=0.0076$ and A2780cisR: $F(10,11) = 7,24$, $P=0.002$)**.** * $P<0.05$. ** $P<0.01$ indicates significance vs. the control.

Supplementary Figure 5

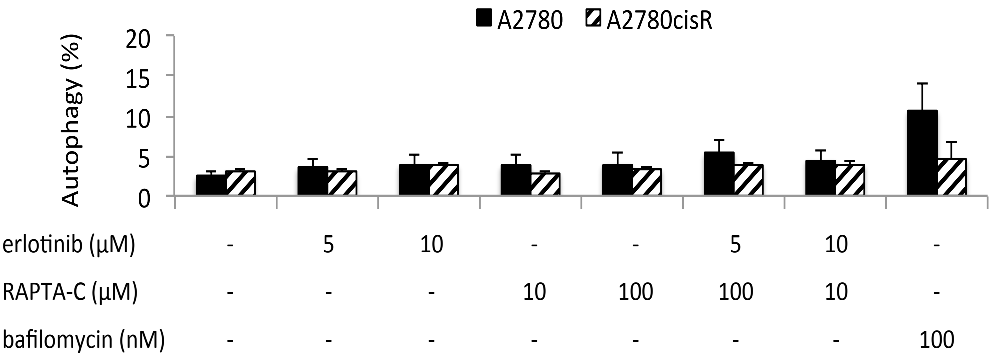

Supplementary Figure 5. Staining of autophagic vacuoles by monodansylcadavarine (MDC) in A2780 and A2780cisR cells. Autophagy was assessed using flow cytometry analysis based on staining of autophagic vacuoles by the fluorescent compound MDC. Bafilomycin was included to serve as negative control. Values represent the mean of 3-4 independent experiments and error bars represent SEM.

Supplementary Figure 6

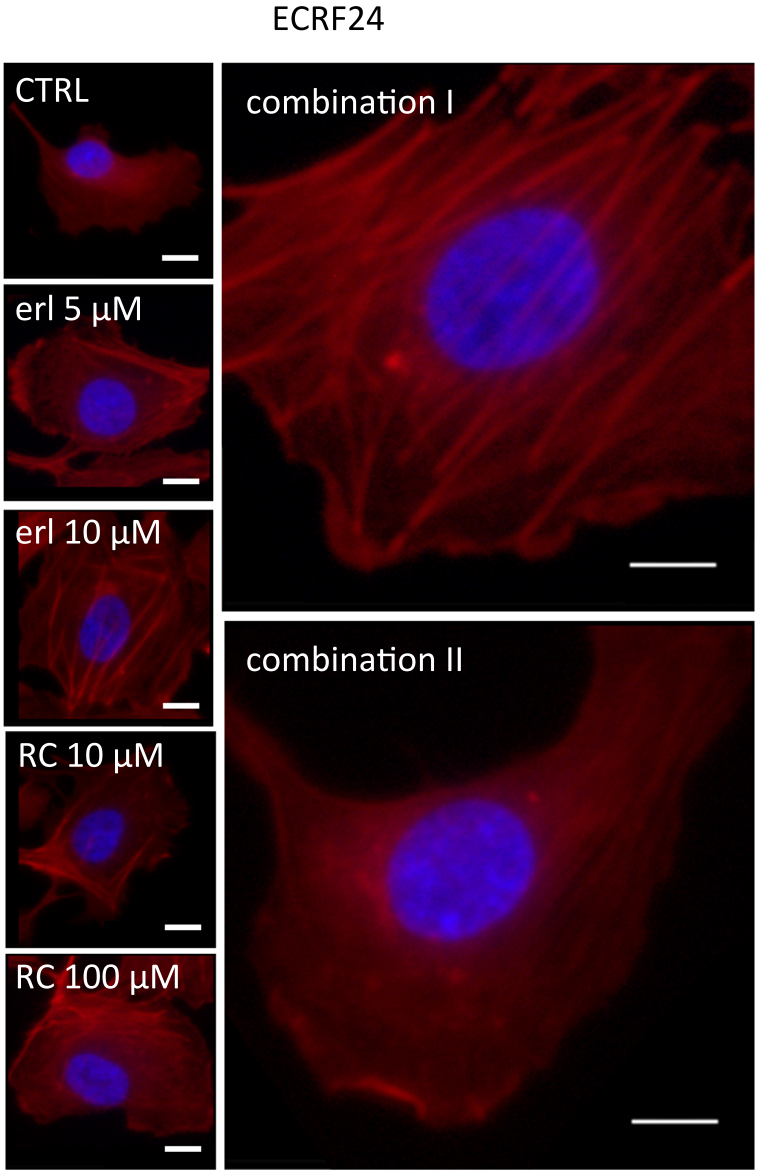

Supplementary Figure 6. Representative images of staining for DAPI (blue) and phalloidin (red) in ECRF24 cells treated with erlotinib, RAPTA-C or their combination. The combination consisting of erlotinib 10 µM/RAPTA-C 10 µM is marked as **I** and erlotinib 5 µM/RAPTA-C 100 µM is marked as **II**.

Supplementary Figure 7

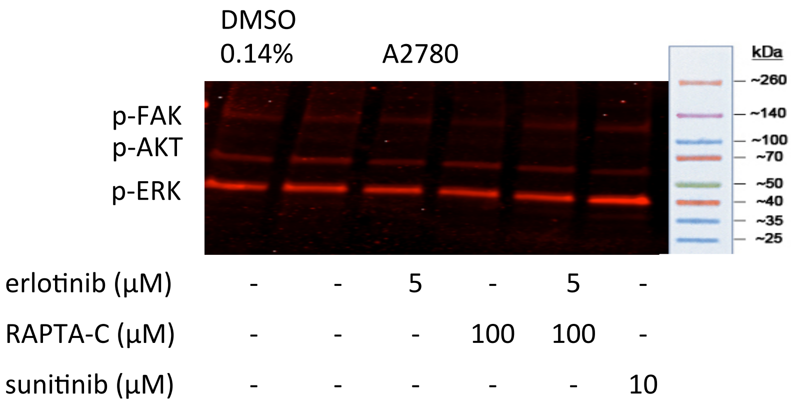

Supplementary Figure 7. Western Blot analysis of erlotinib / RAPTA-C treatment in A2780 cells. Cells were treated for 2 h after which cell lysates were prepared. Immunoblotting was performed using phosphorylated Akt, phosphorylated Erk and phosphorylated FAK antibodies.

Supplementary Figure 8

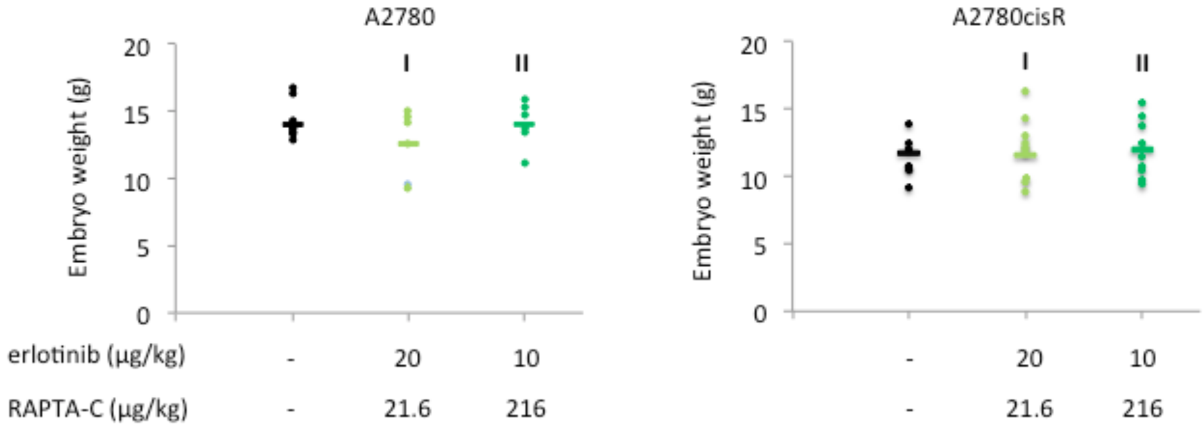

Supplementary Figure 8. Embryo weight in A2780 and A2780cisR tumor models implanted on the CAM. Embryo weight (g) was measured at treatment day 8 when the experiment was terminated (one-way ANOVA: $F(2, 21)=1.23$, $P=0.311$ for the A2780 model and $F(2,28) = 0.087$, $P=0.917$ for the A2780cisR model).
